# The Transcriptional Cell Atlas of Testis Development in Sheep at Pre-Sexual Maturity

**DOI:** 10.3390/cimb44020033

**Published:** 2022-01-19

**Authors:** Yi Wu, Tingting Guo, Jianye Li, Chune Niu, Weibo Sun, Shaohua Zhu, Hongchang Zhao, Guoyan Qiao, Mei Han, Xue He, Zengkui Lu, Chao Yuan, Jianlin Han, Jianbin Liu, Bohui Yang, Yaojing Yue

**Affiliations:** 1 Lanzhou Institute of Husbandry and Pharmaceutical Sciences, Chinese Academy of Agricultural Sciences (CAAS), Lanzhou 730050, China; wy1593050417@126.com (Y.W.); guotingting@caas.cn (T.G.); lijianye218@163.com (J.L.); chuneniu@163.com (C.N.); swb887246@126.com (W.S.); zhu87932890@outlook.com (S.Z.); 18837101296@163.com (H.Z.); qhqiaogy@163.com (G.Q.); 82101186189@caas.cn (M.H.); luzengkui@caas.cn (Z.L.); yuanchao@caas.cn (C.Y.); liujianbin@caas.cn (J.L.); 2 College of Biological Sciences, Northwest Minzu University, Lanzhou 730050, China; 15293100105@163.com; 3 CAAS-ILRI Joint Laboratory of Livestock and Forage Genetic Resources, Institute of Animal Science, Chinese Academy of Agricultural Sciences (CAAS), Beijing 100193, China; h.jianlin@cgiar.org; 4 Livestock Genetics Program, International Livestock Research Institute (ILRI), Nairobi 00100, Kenya

**Keywords:** scRNA-seq, testis, spermatogenesis, sheep

## Abstract

Sheep testes undergo a dramatic rate of development with structural changes during pre-sexual maturity, including the proliferation and maturation of somatic niche cells and the initiation of spermatogenesis. To explore this complex process, 12,843 testicular cells from three males at pre-sexual maturity (three-month-old) were sequenced using the 10× Genomics Chromium^TM^ single-cell RNA-seq (scRNA-seq) technology. Nine testicular somatic cell types (Sertoli cells, myoid cells, monocytes, macrophages, Leydig cells, dendritic cells, endothelial cells, smooth muscle cells, and leukocytes) and an unknown cell cluster were observed. In particular, five male germ cell types (including two types of undifferentiated spermatogonia (A_pale_ and A_dark_), primary spermatocytes, secondary spermatocytes, and sperm cells) were identified. Interestingly, A_pale_ and A_dark_ were found to be two distinct states of undifferentiated spermatogonia. Further analysis identified specific marker genes, including *UCHL1*, *DDX4*, *SOHLH1*, *KITLG*, and *PCNA*, in the germ cells at different states of differentiation. The study revealed significant changes in germline stem cells at pre-sexual maturation, paving the way to explore the candidate factors and pathways for the regulation of germ and somatic cells, and to provide us with opportunities for the establishment of livestock stem cell breeding programs.

## 1. Introduction

Sexual maturity refers to the period after puberty when the male’s body and reproductive organs are further developed, reproductive function is improved, and normal fertility is achieved [1]. During this period, the testes are involved in major physiological changes, including a large increase in their volume and the initiation of somatic cell proliferation/maturation and spermatogenesis [2]. Spermatogenesis is a process of sperm formation that is highly regulated by multiple factors [3,4,5]. Spermatogonial stem cells (SSCs) are located on the basement membrane of the seminiferous tubules in testes and have the ability to self-renew and differentiate into spermatogenic cells at different developmental stages [6,7,8]. To further explore the mechanism of regulation of SSC proliferation and differentiation, and capture the different cell types involved in spermatogenesis, previous studies have been effective in using laser capture microdissection to obtain a high purity target cell population from the paraffin-embedded tissues of humans [9], cattle [10], and rats [11], but problems have been encountered with nucleic acid contamination and low capture efficiency [12,13]. Conversely, single-cell RNA-seq (scRNA-seq) technology has a high cell capture rate, superior sensitivity, and data accuracy, which better reflect cellular heterogeneity [14].

Until now, scRNA-seq technology has been successfully used in the fields of early embryonic development [15,16,17], organ development [18,19], and tumor therapy [20] in animals and humans. The exploration of cell heterogeneity and the discovery of new cell types and marker genes using scRNA-seq technology have been achieved, leading to a deeper and more comprehensive understanding of life science. In the human testes during puberty, four germ cell clusters were identified according to the expression patterns of marker genes, namely, slow self-renewal and undifferentiated spermatogonia, differentiated spermatogonia, meiotic spermatocytes, and spermatids. In addition, six somatic cell types of the testes, including Sertoli cells, myoid cells, Leydig cells, smooth muscle cells, macrophages, and endothelial cells [21], have been identified. Although scRNA-seq technology has been successfully used to construct a map of human spermatogenesis [22,23,24], little research has been performed on the cellular characteristics and regulatory mechanisms of spermatogenesis in domestic animals such as sheep. In only one previous study, the testes of an adult (1.5-year-old) ram were found to contain five germ cell types, two testicular somatic cell types, and an unknown type of somatic cell [25]. However, undifferentiated spermatogonial cells, essential for long-term spermatogenesis, were not captured, and so the underlying mechanisms regulating spermatogonial cell self-renewal/differentiation and spermatogenesis remain unclear.

In the present study, a total of 12,843 cells from the testes of three pre-sexually mature (three-month-old) sheep were captured for sequencing by using 10× Genomics Chromium^TM^ scRNA-seq technology. Nine testicular somatic cell types and five male germ cell types were identified. Especially, the A_pale_ and A_dark_ of sheep were found to be two distinct states of undifferentiated spermatogonia in our research. Our results could provide new in-sights for sheep spermatogenesis and spermatogenic cell development and the fundamental information for the male reproductive physiology of livestock.

## 2. Materials and Methods

### 2.1. Materials

#### Experimental Animals and Preparation of Cell Suspensions

Three three-month-old healthy male Hu sheep, maintained at the Gansu Qinghuan Sheep Breeder Co. LtD in Huan county, China, were castrated. After castration, the testicles were collected in Hanks’ balanced salt solution (HBSS), supplemented with 100 IU mL^−^^1^ penicillin–streptomycin at 4 °C, then digested using a two-step enzymatic digestion process within 3 h. The testicular tissue was cut lengthwise, and the internal tissue was washed 5 times with Dulbecco’s phosphate-buffered saline (DPBS), then placed in a small Petri dish. Each tissue sample was shredded using blunt-edged forceps at 4 °C. About 400 milligrams of tissue were taken from the center region of each experimental animal’s testes in a 50 mL centrifuge tube, with 20 mL of 0.25 mg/mL of collagenase type IV and 1 mL of 7 mg/mL DNase I. The tissue was gently shaken and incubated for 5–10 min (but no longer than 10 min) in a water bath at 37 °C, and the tubules were then centrifuged at 50× *g* for 1 min at 4 °C. The gravity-settled tubules were then digested in 10 mL of 0.25% trypsin/ethylenediaminetetraacetic acid (EDTA) and 1 mL of 7 mg/mL DNase I for 25 min at 37 °C. The digestion was stopped by the addition of 1.5 mL DMEM/F-12 of 10% fetal bovine serum (FBS; Gibco, Waltham, MA, USA) and 0.5 mL of 7 mg/mL DNase I. Single testicular cells were obtained by filtration through a cell strainer (70 μm). The cells were resuspended in 2 mL DMEM/F-12 after centrifugation at 400× *g* for 5 min at 4 °C. Finally, the cells were resuspended in DMEM/F-12 with 0.1% bovine serum albumin (BSA; Gibco, Waltham, MA, USA). Cell number and viability were evaluated using a hemocytometer. A cell suspension of ~1000 cells/µL and viability >85% was used for scRNA-seq.

### 2.2. Methods

#### 2.2.1. RNA Extraction and PCR Amplification

The total RNA of single cell suspension were extracted using TRIzol Reagent (Invitrogen, Carlsbad, CA, USA), and the complementary DNA (cDNA) was synthesized using reverse transcription (RT) reagent (Takara, Dalian, Liaoning, China), following the kit instructions. The RNA was then amplified using PCR as follows: 95 °C for 8 min, then 30 cycles of 95 °C for 20 s, 65 °C for 35 s, and 70 °C for 30 s, after which the RNA was denatured at 90 °C for 20 s, 55 °C for 1 min, then stored at 4 °C. A 1.5% agarose gel electrophoresis was used to detect the integrity of 28S and 18S bands, as well as their ratio; the concentration was determined using a ThermoScientific NanoDrop 2000c (ThermoFisher Scientific Inc., Waltham, MA, USA). Only samples with 1.8 < OD 260/280 nm < 2.0, OD 260/230 nm ≥ 2.0, RNA Integrity Number: RIN ≥ 6.5, and 28S/18S ≥ 1.0 were used for subsequent analyses, and the PCR primers are listed in Supplementary Table S1.

#### 2.2.2. Single Testicular Cell RNA-seq Performance, cDNA Library Construction, and Sequencing

After enzymatic dissociation, we used a mouth pipette to immediately transfer single testicular cells into a prepared lysis buffer with an 8-nt barcode. Briefly, each sample was vortexed for 45 s and incubated at 65 °C for 5 min to release the RNAs. Then, the first-strand cDNA was reverse-synthesized using oligo (dT) and template-switching oligonucleotides (TSO). The synthesized first-strand cDNAs were amplified by 16 cycles. All reactions were performed in the original tubes. Subsequently, the products of single cells were pooled and purified. Next, 20 ng of cDNA was used as the template for PCR with the IS primer and Biotin index primer for an additional 4 cycles. Finally, each amplified cDNA had a unique index labeled with a biotin tag. Approximately 100 ng of cDNA was then sheared with Covaris S2 to obtain fragments with an average length of approximately 300 bp. Dynabeads MyOne Streptavidin C1 beads (Thermo Fisher) were used to enrich the 3’-terminal cDNAs. Sequencing was performed on Illumina NovaSeq 6000 System by Shanghai Oyi Biomedical Technology Co., Ltd. (Shanghai, China).

#### 2.2.3. Mapping, Cell Identification, and Cluster Analysis

Firstly, Cell Ranger software was used to further conduct quality control on the experimental data to eliminate multicellular aggregates, cell doublets, or cells that were unencapsulated. The corresponding Read 2 sequences were mapped to the domestic sheep reference genome (*Ovis aries*, Oar_4.0) by STAR software to determine the source genes of the reads that completed gene expression quantification. The Seruat package was used to screen the cells following preliminary quality control, while principal component analysis (PCA) for dimensionality reduction was used to analyze the similarity between qualified cells. The filtering criteria for viable cells included the number of genes per cell, UMI values within ±2 standard deviation, and a mitochondrial gene proportion of less than 5%. These criteria were considered indicative of high-quality cells for downstream analyses. Cells that had similar gene expression profiles were clustered together to represent a cell population, according to the gene expression levels of each cell. Uniform manifold approximation and projection (UMAP) was used to visualize the single cell clustering of the top 15 principal components in the PCA dimensionality reduction evaluation. A mutual nearest neighbor (MNN)-clustering algorithm with a resolution of 0.2 was used for clustering and classifying the cell types.

#### 2.2.4. Differential Expression of Genes

The differentially expressed genes (DEGs) in each cluster were identified to explore the marker genes of each cell type by screening their expression levels based on fold changes and *p*-values. Genes highly expressed in a specific cell cluster are generally used as markers to identify that cell type. In the present study, the differential gene expression was calculated using Seurat software. To enhance the accuracy and efficiency of analysis, DEGs were also considered marker genes where their expressions were observed in at least 10% of the cells in a cluster with |log fold change| > 0.25.

#### 2.2.5. Functional Enrichment Analysis of Marker Genes

To study the differences between each cell types, we performed enrichment analysis of DEGs of each cell type in DAVID (https://david.ncifcrf.gov/, last accessed on 20 November 2021) and GOATOOLS (https://github.com/tanghaibao/goatools, last accessed on 20 November 2021). The functional enrichment of DEGs was analyzed using gene ontology (GO) and the Kyoto Encyclopedia of Genes and Genomes (KEGG). The GO database was used to classify and annotate genes and gene products in terms of biological process (BP), molecular function (MF), and cellular component (CC). The enrichment ratio was calculated, and the 10 most enriched GO and KEGG terms were recorded.

#### 2.2.6. Cell Trajectory Analysis

Cell trajectory analysis calculated the inter-cell gene expression trajectory through the differential expression patterns of key genes, predicting changes in cells over time, and thus simulating changes in cell expression levels. Cell trajectory analysis was performed in male germ cells using the Monocle 2 package (v2.16.0).

#### 2.2.7. Histological Observation and Immunohistochemical Analysis

Samples (1.5 cm^3^) of testicular tissue were collected and placed in Petri dishes with HBSS or DMEM/F-12 and then fixed with 4% paraformaldehyde (PFA) for 48 h at 4 °C, replacing the PFA once during the incubation. The tissue was then dehydrated and embedded, after which 4–6 μm-thick sections were cut and stained with hematoxylin and eosin for histological analysis. Additionally, the expression and localization of a number of marker genes were identified using immunohistochemical analysis using the following primary antibodies: rabbit anti-cyclin A1 (1:100 dilution, ab133183; Abcam, Cambridge, UK), rabbit anti-UCHL1 (1:50 dilution, bs-11677R; Boosen, Beijing, China), and rabbit anti-SCF (1:100 dilution, ab64677; Abcam, Cambridge, UK). Goat anti-rabbit IgG (1:50 dilution, SP-9001; Jin Qiao, Beijing, China) was used to catalyze the conversion of DAB to a brown precipitate during immunohistochemical staining. Testis morphology was then evaluated using 100× and 400× magnifications using a digital tricamera microscope.

## 3. Results

### 3.1. Overview of scRNA-seq Data in Sheep Testes

The testis is the most complex organ of the transcriptome and expresses more than 80% of the protein-coding genes in humans and other species [26,27]. The complexity of the testicular transcriptome is closely related to spermatogenesis, which is a complex and continuous process that occurs asynchronously in the seminiferous tubules. To study the cellular composition of testicular tissue, we obtained the single cell suspension with more than 85% cell viability from male sheep (Supplementary Figure S1). In addition, germ and somatic cells within the testicular tissue sections were observed at the various stages (Supplementary Figure S1). The expressions of known marker genes in the testicular tissue and the tissue derived RNA were evaluated after PCR amplification, as displayed in Supplementary Figure S2, including the reference genes of *GAPDH*, *VWF*, *NOTCH3*, *IGF1*, *ACTA2*, *SOX9*, *CD83*, *PENK*, *S100B*, *BEX4*, *PEGFRB*, *NR6A1*, *ID4*, *CDH11*, *DAZL*, *BCAT2*, *PCNA*, *KITLG*, *DDX4*, *TOP2A*, *UCHL1,* and *SOHLH1*. These genes were expressed in the testicular tissue. The results confirmed the integrity of the testicular cell types obtained.

Single testicular cells were also encapsulated for 10× Genomics scRNA-seq data, as shown in Supplementary Figure S2. A total of 12,843 testicular cells were sequenced, of which 12,546 were analyzed in accordance with the filtering settings. A total of 92.9% reads were mapped to the sheep reference genome. The mapping had a high degree of confidence in 84.5% of the reads. In addition, a Q score of 30 (Q30) was achieved in 96.1, 92.8, and 95.8% of the reads of the bases in the barcodes, RNA, and UMI values, respectively. In total, 22,033 genes were analyzed, with a mean of 1338 genes identified in each cell (Supplementary Table S2).

### 3.2. Identification and Cluster Analysis of Testicular Cell Types

**Figure 1:**
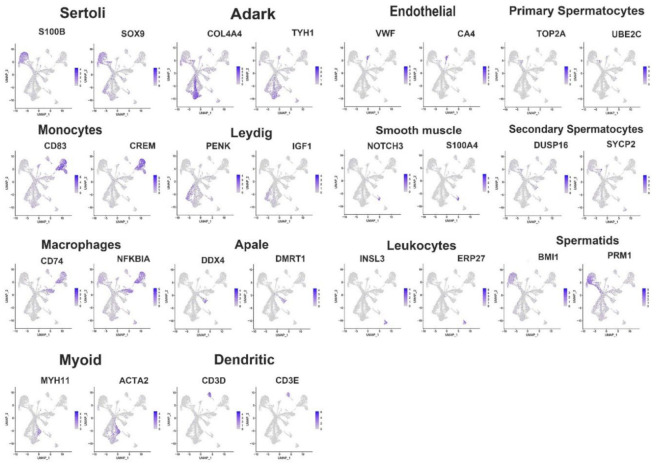
Sheep testicular cell classification and cell type identification. The expression patterns of the known marker genes projected on the UMAP plot. The colors from blue to gray represent the expression levels from high to low.

The scRNA-seq of 12,843 cells yielded 150 Gb of data. Using a number of software packages, including Cell Ranger, Seurat, PCA, and UMAP, the sheep testicular cells were identified and subjected to cluster analysis. As shown in Figure 1, 15 clusters were identified. The literature was searched and the cell marker database (http://biocc.hrbmu.edu.cn/CellMarker/, last accessed on 20 November 2021) was used to identify the cell types in each cluster. Besides, we tested the expression localization of known marker genes in our cells and further predicted the cell types (Figure 1). Clusters 5, 7, and 12–14 were defined as male germ cells, with cluster 5 representing A_dark_ (*THY1*), cluster 7 consisting of A_pale_ (*UCHL1*), cluster 12 identifying primary spermatocytes (*EML5*), cluster 13 defined as secondary spermatocytes (*MME*), and cluster 14 considered as spermatids (*PHGDH*). Clusters 1–4, 8–11, and 6 were defined as Sertoli cells (*SOX9*), myoid cells (*EPHA3*), monocytes (*CD83*), macrophages (*ACP5*), Leydig cells (*EBF1*), dendritic cells (*CD3E*), endothelial cells (*VWF*), smooth muscle cells (*NOTCH3*), and leukocytes (*LHCGR*), respectively (see Supplementary Figure S3 for additional markers). There was also one cell cluster for which the type was unknown. In addition, the numbers of each cell type were counted, as detailed in Supplementary Table S4. The number of Sertoli cells was greater than other somatic cell types, and there were large numbers of A_dark_ and A_pale_, but no significant difference in the number of other germ cells.

### 3.3. DEG Analysis of Sheep Germ and Somatic Cells

**Figure 2:**
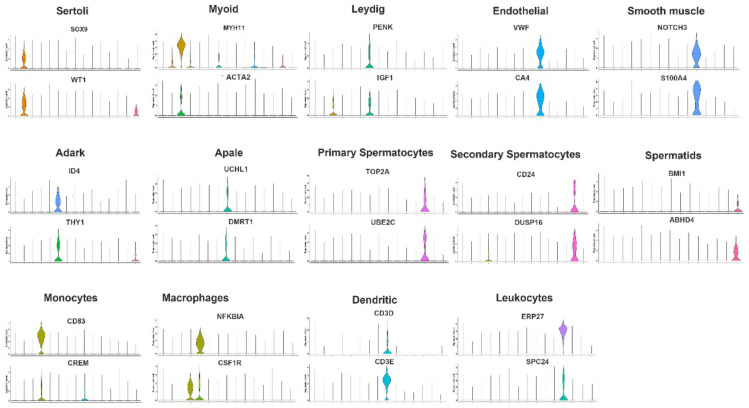
Marker gene identification of each testicular cell type. Expression patterns (violin plot) of each cell-specific gene across the different clusters.

In the male germ cell populations, there were 613, 675, 649, 1254, and 422 marker genes in Apale spermatogonia, Adark spermatogonia, primary spermatocytes, secondary spermatocytes, and spermatids, respectively. There were 1488, 833, 1454, 684, 848, 1662, 717, 621, and 636 DEGs in the Sertoli cells, myoid cells, monocytes, macrophages, Leydig cells, dendritic cells, endothelial cells, smooth muscle cells, and leukocytes, respectively. The 100 most highly expressed cell markers of male germ and somatic cells are listed in Supplementary Table S3. To further determine the expressions of the marker genes in the different clusters, two genes from each cluster were selected for marker gene visualization. The results demonstrated that marker gene expressions in the different clusters were specific (Figure 2).

### 3.4. Functional Enrichment Analysis of Sheep Testicular Cells

#### 3.4.1. Germ Cells

Spermatogenesis is a process of sperm formation that is highly regulated by multiple factors. To study the differences between the cell types, the marker genes of each germ cell cluster were annotated using the GO and KEGG databases. The marker genes in A_pale_ and A_dark_ were significantly enriched in protein folding, spermatogenesis, the binding of sperm, and the positive regulation of cell cycle. For the CC, marker genes were enriched in the cell body, actin cytoskeleton, and cell architecture when the protein complexes in differentiated spermatogonia were compared. For the primary spermatocytes, secondary spermatocytes, and spermatids, the marker genes mainly represented those responsible for sexual reproduction, sperm cell development, sperm cell differentiation, spermatogenesis, and male gametogenesis.

**Figure 3:**
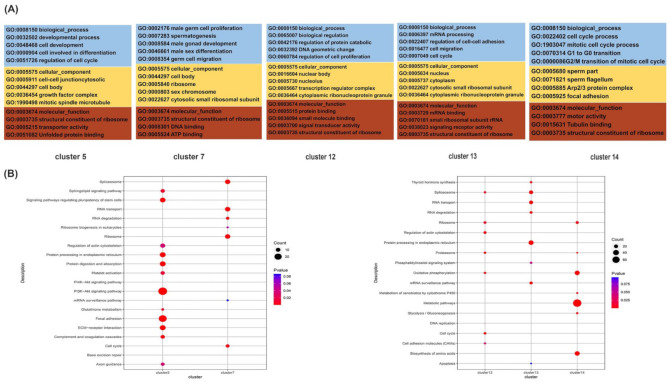
Functional enrichment analysis of testicular germ cell types. (**A**) The top GO analysis of five germ cell types. (**B**) The top KEGG analysis of five germ cell types.

For the MF, the marker genes of spermatocytes and spermatids were involved in DNA binding, NAD activity, and protein binding, while the marker genes of undifferentiated spermatogonia were enriched in ATP binding, RNA transport, ribosomes, and the cell cycle (Figure 3A and Supplementary Table S5). Pathway enrichment demonstrated that the genes of the A_pale_ and A_dark_ clusters were principally enriched in the spliceosome, regulation of the actin cytoskeleton, signaling pathways regulating the pluripotency of stem cells, RNA transport, and the PI3K-Akt signaling pathway, while the majority of genes of spermatocytes and spermatids were involved in the activin as candidate key players during the latency period (Figure 3B). In conclusion, the germ cells are mainly concentrated in GO and KEGG terms related to transcription, gonadal development, and germ cell migration in male animals, further illustrating the proliferation and differentiation of germ cell in the pre-sexual period.

#### 3.4.2. Somatic Cells

**Figure 4:**
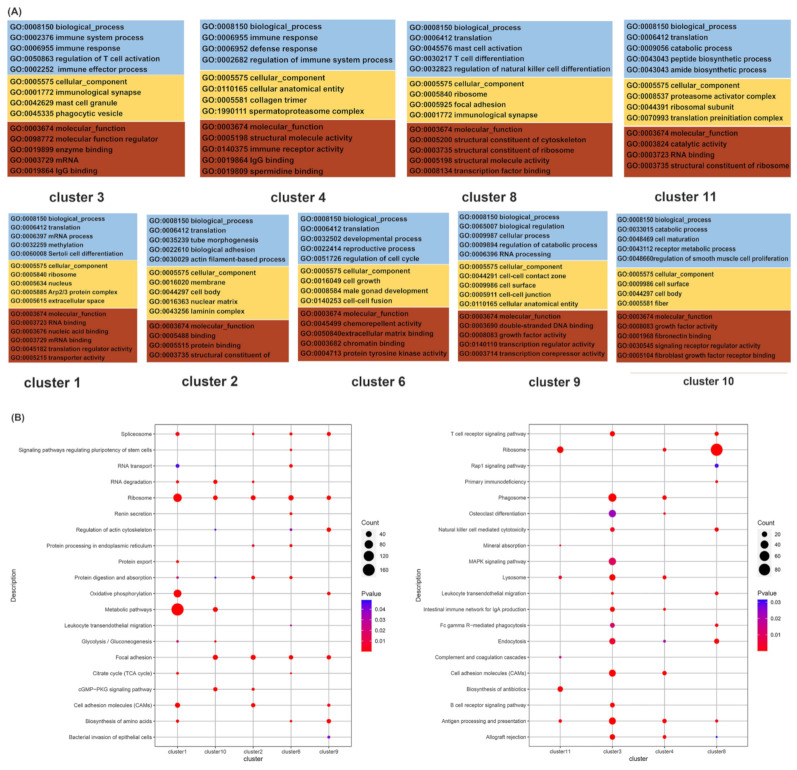
Functional enrichment analysis of testicular somatic cell types. (**A**) The top GO analysis of three somatic cell types. (**B**) The top KEGG analysis of three somatic cell types.

Somatic cells in the testes are instrumental in spermatogenesis; we performed the GO and KEGG analyses of marker genes to investigate the different functions of somatic cells. GO analysis demonstrated that translation, the oxidation–reduction process, rRNA processing, and cellular amide metabolic processes were significantly enriched in the BP in all types of somatic cells. While mitochondria, extracellular exosomes, ribosomes, nucleoli, the Arp2/3 protein complex, and the small ribosomal subunit were enriched in CC, the structural constituent of ribosomes, rRNA binding, mRNA binding, and translation initiation factor activity were significantly enriched in MF. The most significantly enriched GO terms of the marker genes of somatic cells are displayed in Figure 4A and Supplementary Table S5, which demonstrates that the marker genes of the Sertoli cells were enriched in the envelope of CC. In addition, the most significantly enriched KEGG pathways in Figure 4B were the ribosome pathway in testicular somatic cells, which indicated that the three-month-old testis cells were transitively active.

### 3.5. Cell Trajectory Analysis of Sheep Testicular Germ Cells

**Figure 5:**
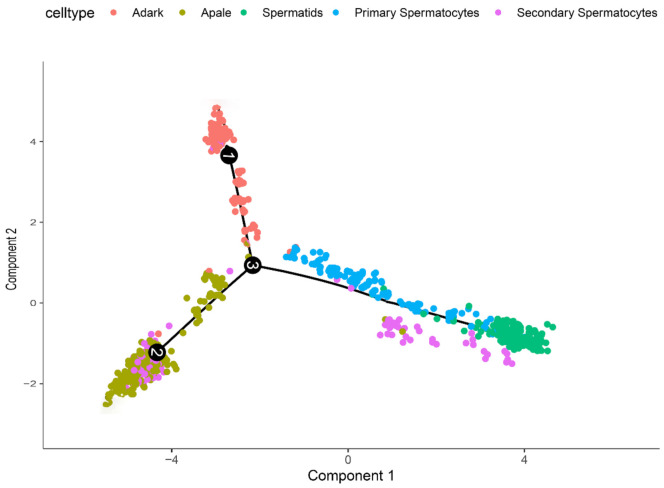
Cell trajectory analysis of sheep testicular germ cells. Cell trajectory analysis of five germ cell types. This analysis indicated that these cell subsets had the following developmental order: A_pale_—primary spermatocytes—secondary spermatocytes—spermatids, while A_dark_ are slow self-propagation.

Spermatogenesis is a complex process involving mitotic cell division, meiosis, and the process of spermiogenesis [28]. In the present study, five germ cell types were included for cluster analysis and they were identified using marker genes, while the developmental process of germ cells was revealed using the Monocle 2 software package. It is worth noting that there are two different starting points for cellular trajectory analysis of germ cells, that is, from undifferentiated spermatogonia to spermatids. The trajectories of the five germ cell types were mapped (Figure 5).

**Figure 6:**
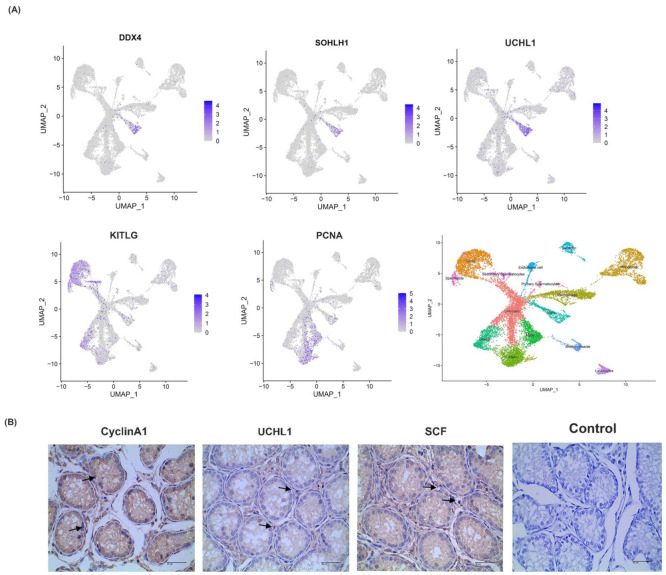
Expression and distribution of several potential genes. (**A**) The expression distribution of DDX4, SOHLH1, UCHL1, KITLG, and PCNA in sheep testicular cells. (**B**) Immunohistochemistry analysis of the expression location of CyclinA1, UCHL1, and SCF in sheep testis. The testis morphology was observed under 400× magnification by a microscope.

To further explore the changes in gene expression within the five clusters, multiple genes were filtered using the Monocle module in R software, with expression levels displayed in Figure 6A. The results indicated that *CDH11*, *UCHL1*, and *DDX4* were highly expressed in undifferentiated spermatogonia, while there was no expression in spermatocyte or spermatids. In contrast, *TOP2A* gene expression was only observed in spermatocytes, the expression levels gradually increasing as meiosis occurred. Immunohistochemical staining also confirmed the expression of these genes in sheep testes (Figure 6B).

## 4. Discussion

The testes of male animals before sexual maturation develop into a compact structure by interacting between somatic cells and germ cells, forming a niche, with spermatogonial differentiation and the regulation of hormone signaling molecules ultimately promoting stable spermatogenesis [5]. Previously, studies of spermatogenesis mostly have focused on mice and humans, with few large animals such as sheep and cattle having been reported [29]. To reveal this complex process in more detail at the molecular level, we used the 10× Genomics scRNA-seq technology to analyze the transcription process of testicular cells in sheep at pre-sexual maturity, and constructed a transcription map of the cells in testes of sheep at pre-sexual maturity. In addition, the scRNA-seq data were submitted to the GEO database. Five male germ cell types at different stages and nine testicular somatic cell types at the sexual maturation stage were clustered, and the marker genes of the different cell types were identified. Spermatogonial stem cells have the ability to self-renew, with differentiation similar to other tissue-specific stem cells [30]. The testes of nonhuman primates and humans contain two morphologically distinct types of undifferentiated spermatogonia, identified as A_dark_ and A_pale_, based on the differences in nuclear morphology and the intensity of hematoxylin staining [31]. The nucleus of A_dark_ spermatogonia exhibited homogeneous, dense, intensively stainable chromatin and at least one chromatin-free cavity (chromatin rarefaction zone), whereas the chromatin of A_pale_ spermatogonia were coarser, less dense, less stainable and devoid of the rarefaction zone. A_pale_ and A_dark_ were distinguishable by their typical nuclei localization (peripheral vs. central) and by the distance of the cells to the tubular basement membrane (close vs. minimal contact) [31,32]. We further observed that *ID4*, *SOHLH1*, *UCHL1*, and *EGR4* genes also appeared to be expressed in all of the undifferentiated spermatogonia clusters (Supplementary Table S3). Fortunately, cells in the clusters five and seven are characterized by expression of *EXOSC10*, *THY1* (A_dark_) and *DMRT1* (A_pale_). Based on gene expression patterns, we thus cautiously infer that cluster five and cluster seven correspond with A_dark_ and A_pale_ spermatogonia. Following the study of specific genes of mammalian testicular development, it has been revealed that their expression differs in different stages of testicular development when compared with the surrounding tissues. However, traditional transcriptome sequencing only provides an overall analysis of testicular tissue, with RNA expression obtained from mixed cell populations, ignoring cell heterogeneity [33].

Five germ cell types in male animals were clustered in the present study, including two undifferentiated spermatogonial stem cells (A_pale_ and A_dark_), primary and secondary spermatocytes, and spermatids. However, when compared with other studies, no mature sperm were captured in the present study, probably because the number of mature sperm in threemonth-old sheep testes is rather low [34]. In previous studies, more than 10% of undifferentiated spermatogonial cells clustered together in sheep aged 3–4 months, the most suitable period for gathering spermatogonial stem cells [35], and had high expression of specific genes. Spermatogonial marker genes, including *DDX4*, *ID4*, *UCHL1*, and *SOHLH1,* were enriched in clusters 5 and 7 in the present study, revealing that spermatogonial cells were present in the testes of three-month-old sheep. Based on differential expression analysis, marker genes that more accurately represented sheep testicular cells were screened, and a key gene for the study of sheep testicular development and spermatogenesis was identified.

Functional enrichment analysis demonstrated that most cells in spermatogonia clusters had a cell cycle gene expression profile indicative of them being in gap 0 (G0) or gap 1 (G1) phase, with only a few cells in synthesis (S) or gap 2 (G2) mitosis phase. This suggested that most spermatogonia were not proliferating and/or undergoing slow proliferation, consistent with past studies that have directly examined the proliferation of undifferentiated human spermatogonia. The marker genes of undifferentiated spermatogonia (A_pale_ and A_dark_) were enriched in the PI3K-Akt signaling pathway, which was activated by many types of cellular stimulation or toxicity, to regulate cell proliferation and apoptosis. Primary spermatocytes, secondary spermatocytes, and spermatids displayed similar functional enrichments, which were mainly involved in cell metabolism, spermatogenesis, and cell differentiation, indicating that these three stages were related to the formation of sperm cells. The results suggested that during spermatogenesis in male animals, starting with undifferentiated spermatogonia, the number of cells was maintained, and they underwent meiosis, differentiating into lower germ cells. In addition, there were two distinct states of undifferentiated spermatogonia from primordial germ cells in the testes, with an entirely different direction of differentiation [32]. A_pale_ spermatogonia were involved in meiosis, producing spermatocytes and representing the normal process of spermatogenesis in male animals, while A_dark_ spermatogonia, considered a “reserve” stem cell, participated in spermatogenesis only in injured animals [32]. This conclusion was further supported by a trajectory analysis of testicular germ cells, which was consistent with the results observed in monkey spermatogonia [36].

The somatic cells of the testes have been categorized as Sertoli cells, Leydig cells, myoid cells, endothelial cells, smooth muscle cells, and immune cells in previous studies [1]. The stability of the spermatogonial stem cell niche is ensured by the interaction of regulatory factors secreted by different somatic cell types of the testes, which determine the fate of SSCs (i.e., self-renewal or differentiation) [37]. In addition, immune cells play an important role in maintaining the stability of testes by regulating and participating in their immune response [38]. In the present study, the marker genes of Sertoli cells, Leydig cells, and myoid cells were mostly enriched in transcription and ribosomal pathways, and they were involved in the formation of proteins with molecular functions. The marker genes of immune cells were involved in RNA molecular function. Although the functions of these somatic cell types in spermatogenesis are relatively well established, little is known about their development and function prior to spermatogenesis, particularly at the molecular level.

To explore the key genes regulating spermatogenesis, a cell trajectory analysis of undifferentiated spermatogonia (A_pale_ and A_dark_) and differentiated spermatogonia (primary spermatocytes, secondary spermatocytes, and sperm cells) was performed, thereby determining the state of germ cell development. Five potential genes, including *UCHL1*, *DDX4*, *SOHLH1*, *KITLG*, and *PCNA*, were identified, displaying different levels of their expressions during the process of spermatogenesis. For example, UCHL1 (PGP9.5), a deubiquitinating enzyme, inhibits protein degradation by reversing ubiquitin modifications [39]. Differences in the localization of UCHL1 play important roles in regulating cell proliferation and differentiation through the post-translational ubiquitination of proteins [39]. Localization of UCHL1 in the cytoplasm is a marker of non-proliferative spermatogonia in primates, and its conservative expression regulates spermatogenesis in mammalian testes [40].

Notably, the *UCHL1* gene is expressed in rodent spermatogonia and Sertoli cells, whereas in other mammals, including humans, non-primates, and domestic animals, it is expressed only in spermatocytes and the spermatogonia of seminiferous tubules [39]. UCHL1 is considered the most appropriate marker of spermatogonia and meiosis in male germ cells, consistent with the result of the present study. The DEAD-box polypeptide 4 (DDX4, also known as VASA) gene encodes an ATP-dependent RNA helicase that is a member of the Asp-Glu-Ala-Asp (DEAD)-box protein family (DDX4) [41]. It has been reported that the *DDX4* gene is expressed in human, pig, and horse germ cells and can promote cell proliferation [42,43,44] while *SOHLH1* gene (spermatogenesis and oogenesis specific basic helix-loop-helix (bHLH) transcription factor) [45] has been shown in previous studies to be preferentially expressed in A_pale_ spermatogonia. The expression levels of *KITLG* increase with the progression of spermatogenesis, and its expression in spermatocytes has been demonstrated following spermatogonial differentiation [46]. Through the study of testicular tissue, we have identified five different types of germ cells and nine types of somatic cells in the testis of Hu sheep at pre-sexual maturity, and the characteristic genes specifically expressed in each cell cluster during spermatogenesis. The DEGs were mainly enriched in Notch, TGF-βsignaling pathways, and the signaling pathway that regulated stem cell pluripotency.

In conclusion, we applied scRNA-seq technology to analyze the process of spermatogenesis in Hu sheep, revealing the types of testicular cells of sheep during pre-sexual maturity. The results of this study should enrich our understanding of the spermatogenesis of Hu sheep and provide theoretical and technical support for sheep breeding research.

## Data Availability

The datasets presented in this study can be found in online repositories. The names of the repository/repositories and accession number(s) can be found below: GEO database, accession no: GSE184343, available at https://www.ncbi.nlm.nih.gov/geo/query/acc.cgi?acc=GSE184343, last accessed on 20 November 2021.

## Supplementary Materials

The following supporting information can be downloaded at: https://www.mdpi.com/article/10.3390/cimb44020033/s1, Figure S1: Summary of 10× Genomics scRNA-seq technology applied to sheep testes. (A) The sequencing pipeline. (B) The cell viability and number of testicular single cell suspensions. (C) Histomorphological analysis of sheep testes; Figure S2: (A) The expressions of *GAPDH*, *ID4*, *CDH11*, *DAZL*, *KITLG*, *DDX4*, *TOP2A*, *UCHL1*, *SOHLH1*, *NR6A1*, *PEGFRB*, *VWF*, *NOTCH3*, *IGF1*, *ACTA2*, *SOX9*, *BEX4*, *CD83*, *CCL5*, *PENK*, and *S100B* genes in the testicular tissues. (B) Quality control of scRNA-seq data; Figure S3: Heat map of marker genes in each cell type. Different colors at the top of the heat map represent different cell types. The left of the heat map shows the symbol IDs of top 5 marker genes in each cell type. The color from yellow to purple shows their expression levels from high to low; Table S1: List of primer sequences used for the amplification of testis specific genes.; Table S2: Statistical analysis of sheep testicular cells by Cell Ranger software; Table S3: The list of top 100 marker genes of each cell cluster; Table S4: The number of different cell types; Table S5: GO analysis of DEGs in each cell cluster by GOATOOLS.
