# Supplementary material for: The Transcriptional Cell Atlas of Testis Development in Sheep at Pre-Sexual Maturity
Source: Curr Issues Mol Biol. 2022 Jan 19;44(2):483–97. doi: 10.3390/cimb44020033 (PMC8929108; doi:10.3390/cimb44020033)
Supplement: Supplementary file 1 [file cimb-44-00033-s001.zip › Supplementary Figure S2.pdf]

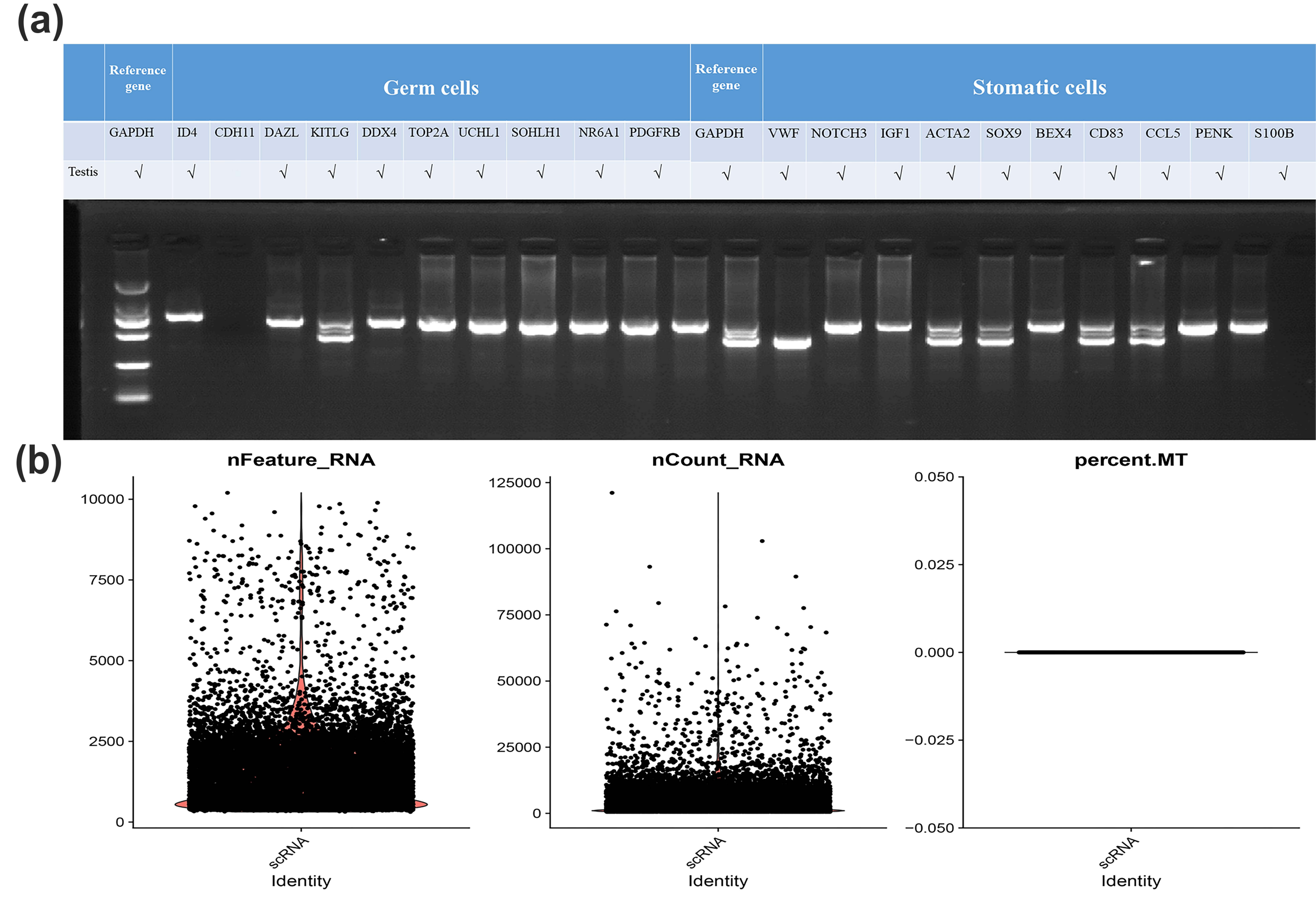

(a)
Reference gene
Germ cells
Reference gene
Stomatic cells
GAPDH
ID4
CDH11
DAZL
KITLG
DDX4
TOP2A
UCHL1
SOHLH1
NR6A1
PDGFRB
GAPDH
VWF
NOTCH3
IGF1
ACTA2
SOX9
BEX4
CD83
CCL5
PENK
S100B
Testis
√
(b)
nFeature_RNA
10000
7500
5000
2500
0
scRNA
Identity
nCount_RNA
125000
100000
75000
50000
25000
0
scRNA
Identity
percent.MT
0.050
0.025
0.000
−0.025
−0.050
scRNA
Identity
