# Supplementary material for: The Transcriptional Cell Atlas of Testis Development in Sheep at Pre-Sexual Maturity
Source: Curr Issues Mol Biol. 2022 Jan 19;44(2):483–97. doi: 10.3390/cimb44020033 (PMC8929108; doi:10.3390/cimb44020033)
Supplement: Supplementary file 1 [file cimb-44-00033-s001.zip › Supplementary Table S1.pdf]

Supplementary Table S1. List of primer sequences used for the amplification of testis specific genes.

Gene symbol	Primer sequence	Product size (bp)
GAPDH	F: GTCAAGGCAGAGAACGGGAA	232
	R: GGTTCACGCCCATCACAAAC	
UCHL1	F: CCTGTGGAACCATCGGACTC	223
	R: CTACCCGACATTGGCCTTCC	
ID4	F: CCTGCAGCACGTTATCGACT	240
	R: AGAGAATGCTGTCGCCCTG	
SOHLH1	F: GGAACTGCACTGCCATCTCTA	113
	R: AATACGTCCACAGTGGGTGG	
DDX4	F: TCCGTCTTGATAACCTCTCCA	258
	R: AGACTTAAGCACTGCCCGAC	
DAZL	F: CCTCTGGAAATGGCCCACAA	183
	R: GACAGAGGGTCAGCAGGTCT	
KITLG	F: GATCGGGTTTGCTTTTGGGG	121
	R: ACTGTTACCCGCCTTATTTCA	
TOP2A	F: AGTCTCCCACCTCCACTACAG	125
	R: TTGCGGCGAGTCTTGGTTTT	
TBX3	F: GTCCTGCTACTGGGGAACAG	220
	R: CACGTTGCTTGATCACTTGGG	
CDH11	F: TGATATCGCCACCCTCCAGA	125
	R: GGGTCATTATCCGCCTCCTG	
VWF	F: TCAGACCTTGGGATGTTGCC	161
	R: TGAATCCGGAGATGACCCCT	
SOX9	F: TCTGGAGACTGCTGAACGAGA	152
	R: AGATGGCGTTGGGCGAGAT	
ACTA2	F: ACGTGGGTGATGAAGCACAA	206
	R: GGTGAGAAGGGTTGGATGCT	
NOTCH3	F: CAGCGAGCACCCTTACTTGA	106
	R: GGTCTGAGCCTGAGCAAGAG	
IGF1	F: TGTACTGTGCGCCTCTCAAG	119
	R: TGTTTCCTGCACTCCCTCTG	
BEX4	F: GACCCCGCTCATCAAAAGGA	206
	R: CGAAGGTGACCTCGATCTGG	
PENK	F: CATTCCGGGAAGTCTGCGAA	106
	R: TGGCATCCTCCTCTGAACCA	
S100B	F: GGAGGGGCAAGAACAGAACT	119
	R: GCGGAAATTGCGATTGGCAT	
CD83	F: CCACAGTGTCATCCAACCCA	220
	R: TGGACAGAAGAGTTCACGGC	
PEGFRB	F: GCACAGCTATGGGATGCTCT	125
	R: CCTGCAAGCTCAAGCAGTTG	
BCTA2	F: GGCTGGAAGGACAACATCTC	183
	R: GACAGAGGGTCAGCAGGTCT	

PCNA	F: CACCAGCGACTCCATCACTA<br>R: AGCGAGCATGTGTCTGTTTG	121
