# Supplementary material for: The Transcriptional Cell Atlas of Testis Development in Sheep at Pre-Sexual Maturity
Source: Curr Issues Mol Biol. 2022 Jan 19;44(2):483–97. doi: 10.3390/cimb44020033 (PMC8929108; doi:10.3390/cimb44020033)
Supplement: Supplementary file 1 [file cimb-44-00033-s001.zip › Supplementary Table S2.pdf]

Supplementary Table S2. Statistical analysis of sheep testicular cells by Cell Ranger software

**Sample**	**Testis**
Estimated Number of Cells	12,843
Mean Reads per Cell	38,730
Median Genes per Cell	1,338
Number of Reads	497,413,101
Valid Barcodes	97.90%
Sequencing Saturation	60.4%
Q30 Bases in Barcode	96.1%
Q30 Bases in RNA Read	92.8%
Q30 Bases in UMI	95.8%
Reads Mapped to Genome	92.9%
Reads Mapped Confidently to Genome	84.5%
Reads Mapped Confidently to Intergenic Regions	13.1%
Reads Mapped Confidently to Intronic Regions	16.2%
Reads Mapped Confidently to Exonic Regions	55.1%
Reads Mapped Confidently to Transcriptome	51.5%
Reads Mapped Antisense to Gene	1.0%
Fraction Reads in Cells	63.5%
Total Genes Detected	22,033
Median UMI Counts per Cell	2,759
