# Supplementary material for: The Transcriptional Cell Atlas of Testis Development in Sheep at Pre-Sexual Maturity
Source: Curr Issues Mol Biol. 2022 Jan 19;44(2):483–97. doi: 10.3390/cimb44020033 (PMC8929108; doi:10.3390/cimb44020033)
Supplement: Supplementary file 1 [file cimb-44-00033-s001.zip › Supplementary Table S4.pdf]

Supplementary Table S4. The number of different cell types

Cell type	Number
A_{dark}	909
A_{pale}	617
Primary spermatocytes	195
Secondary spermatocytes	190
Spermatids	152
Sertoli cells	1678
Myoid cells	1671
Leydig cells	858
Endothelial cells	318
Smooth muscle cells	284
Dendritic cells	319
Macrophages	1232
Monocytes	1572
Leukocytes)	253
Unknown	2298
