# Supplementary figures and images for: The Transcriptional Cell Atlas of Testis Development in Sheep at Pre-Sexual Maturity
Source: Curr Issues Mol Biol. 2022 Jan 19;44(2):483–97. doi: 10.3390/cimb44020033 (PMC8929108; doi:10.3390/cimb44020033)

(A)

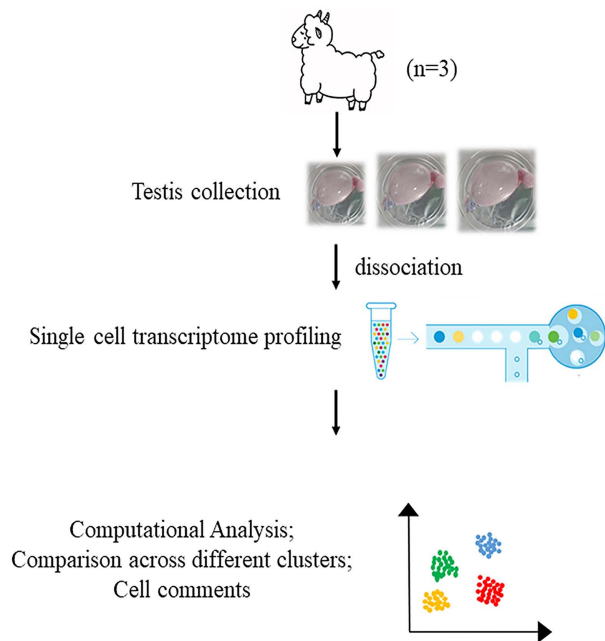

(B)

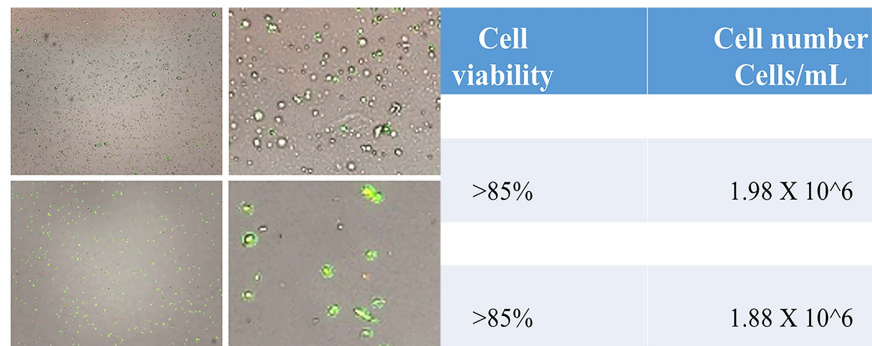

(C)

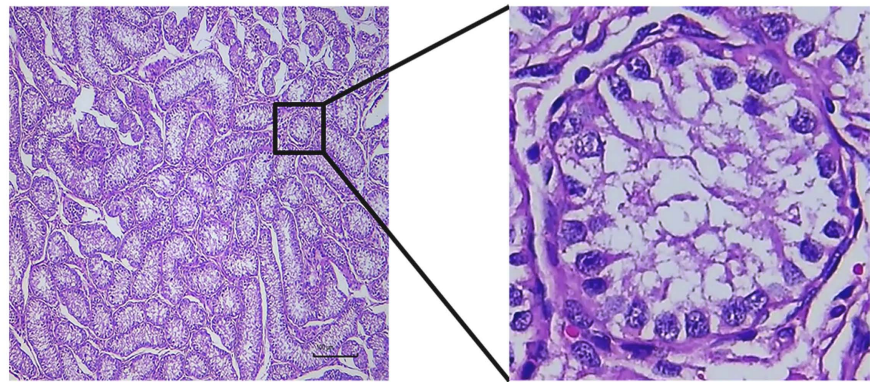

Supplement: Supplementary file 1 [file cimb-44-00033-s001.zip › Supplementary Figure S1.pdf]

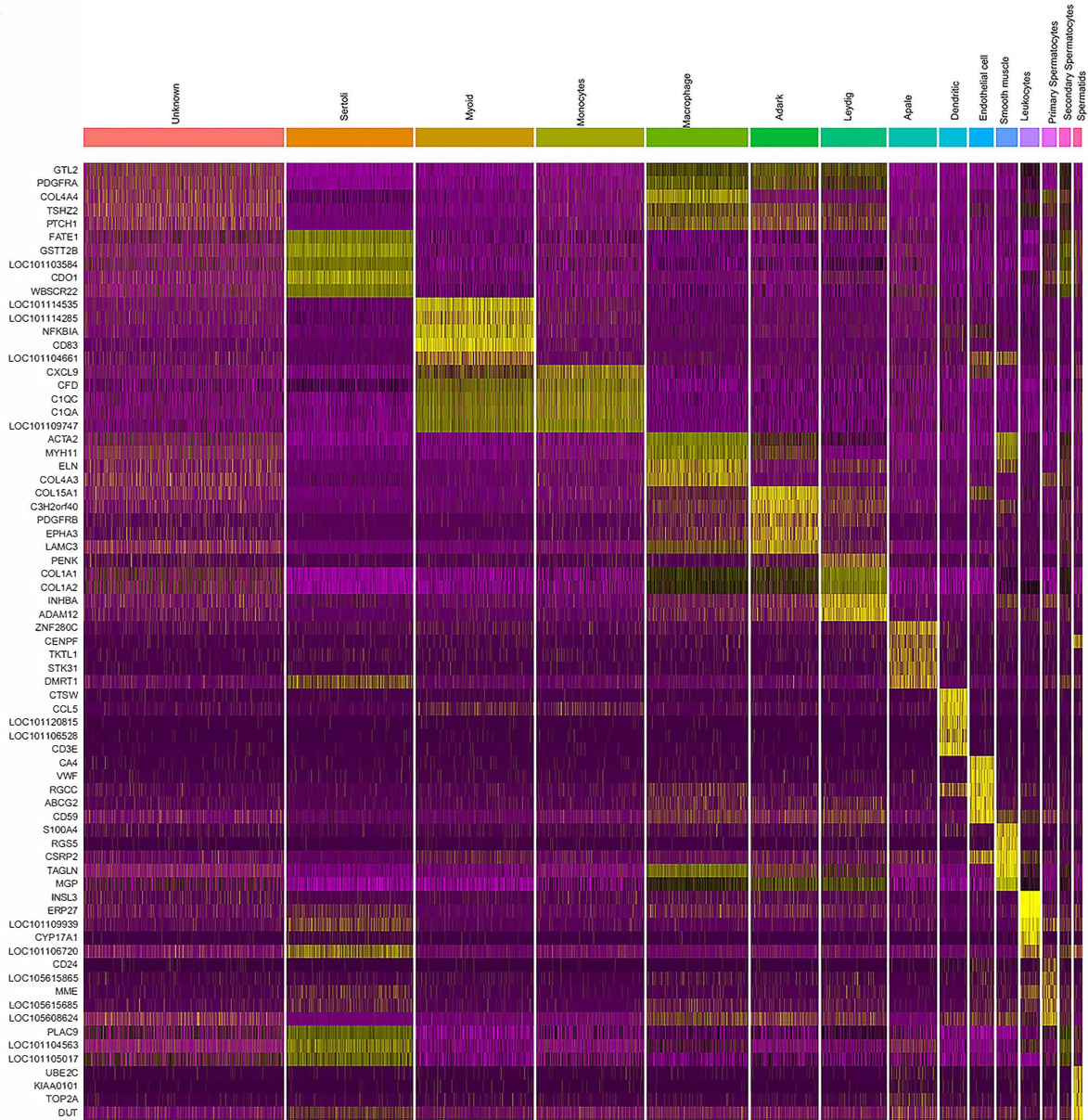

Supplement: Supplementary file 1 [file cimb-44-00033-s001.zip › Supplementary Figure S3.pdf]
